# Eyelid Dynamics Characterization with 120 GHz mmW Radar

**DOI:** 10.3390/s24237464

**Published:** 2024-11-22

**Authors:** Dominik Patscheider, Ruochen Wu, Antoni Broquetas, Albert Aguasca, Jordi Romeu

**Affiliations:** 1 Department of Electrical Engineering and Information Technology (ETIT), Karlsruhe Institute of Technology (KIT), Campus Süd, Engesserstraße 13, 76131 Karlsruhe, Germany; dominik.patscheider@alumni.kit.edu; 2 CommSensLab, Department of Signal Theory and Communications, Universitat Politècnica de Catalunya (UPC), Campus Nord, Carrer de Jordi Girona, 1–3, 08034 Barcelona, Spain; ruochen.wu@upc.edu (R.W.); alberto.aguasca@upc.edu (A.A.); jordi.romeu-robert@upc.edu (J.R.)

**Keywords:** millimeter wave radar, eyelid detection, eye activity, stress, radar beam

## Abstract

This paper presents a new approach to measuring eyelid movement using millimeter wave (mmW) radar technology. A two-step method is proposed, involving the observation of a small resolution cell corresponding to the monitored eye and the evaluation of the phase evolution over the measurement period. Simulations are conducted to support radar system optimization and data interpretation with a focus on detecting eyelid movement patterns and compensating for interference from other parts of the body. The feasibility of using this method with eyeglasses is also explored. The proposed technique’s advantages and limitations are discussed in comparison with existing measurement alternatives. The characteristics of eyelid dynamics, including blink frequency, regularity, duration, and velocity can be used to assess neurological conditions and driver drowsiness.

## 1. Introduction

Millimeter wave radar (mmW radar) technology, which uses electromagnetic waves with wavelengths in the millimeter range, has a number of applications, including estimation of the range and velocity of objects [1,2,3], beam forming for location detection [4], meteorology [5], geodesy [6], air traffic control [7], and seafaring [8]. In this paper, the use of mmW radar for biomedical applications, specifically measuring eye blink rate, is explored. The ability to remotely observe the micrometric deformation of objects is utilized to investigate the characterization of eye blinking dynamics as a potential tool for detecting neurological disorders [9]. This research includes optimization of the radar system, electromagnetic modeling, and experimental evaluation. Eye blinking is a reflex response triggered by a corneal stimulus that helps keep the cornea clean and healthy by stimulating the secretion of mucin and meibum, which are components of the tear film [10]. Studies have categorized eye blinking as spontaneous, reflex, and voluntary [11]. Factors such as stress can affect blink rate [12] and can be used to determine one’s fitness for tasks such as driving. In order to accurately measure eyelid movement and gain sufficient data to understand the dynamics of blinking, this research will employ a two-step approach using Frequency-Modulated Continuous Wave (FMCW) radar. This method aims to overcome the limitations of previous techniques, such as high-speed cameras and magnetic coils, which have difficulty in certain lighting conditions and require significant computational effort to extract the parameters of interest. For a successful evaluation of blinking with regard to specifying neurological disorders, it is crucial to detect the inter-blink interval (IBI), the duration of a blink, and the dynamics of a blink (i.e., the duration and speed of opening and closing the eyelids and the duration of the eye being closed).

## 2. Theoretical Background

A RADAR (RAdio Detection And Ranging system) uses radio waves to detect and measure the distance and velocity of objects [13,14]. In order to achieve accurate measurements, the radar’s beamwidth and distance resolution must be considered. To achieve the required micrometric displacement precision using the FMCW radar, the spectrum of the radar receiver output at the baseband is used to obtain a high-range resolution and the target coarse range estimation. From a sequence of periodic observations, the phase evolution of the target resolution cell provides a very precise submillimetric range, allowing us to estimate eyelid dynamics.

The FMCW radar system typically consists of a waveform generator that produces a voltage to control the frequency of the transmitted signal, which is usually a sawtooth or triangle frequency deviation. The transmitted saw tooth signal can be expressed as

(1) $$s\left(t\right)=A\cos(2\pi f_{0}t+\theta \left(t\right)+\phi_{0}),$$

with the amplitude *A*, the center frequency of the bandpass signal $f_{0}$, the quadratic phase θ(t) producing a linear frequency sweep Δf, and $\phi_{0}$ as the initial phase. For a positive or negative linear FM slope, the phase is

(2) $$\theta \left(t\right)=\pm \pi \mu t^{2},$$

with $\mu =\frac{\Delta f}{T_{M}}$ being the modulation slope within $-\frac{T_{M}}{2}\leq t\leq \frac{T_{M}}{2}$. For a static target, the echo signal received is

(3) $$\begin{array}{c} s_{r}\left(t\right)=\alpha A\cos\left[\omega_{0}t-\omega_{0}\tau +\theta \left(t-\tau \right)+\phi_{0}\right], \end{array}$$

with a signal-level drop α and a target delay τ.

Mixing the signal received with the signal transmitted using a single mixer, or, equivalently, the In-Phase output of an I/Q mixer, results in the beating tone $s_{b}\left(t\right)$ with a beating frequency $f_{b}\left(t\right)$, which is the difference between the transmitted signal and echo-instantaneous frequencies

(4) $$\begin{array}{c} f_{b}\left(t\right)=f\left(t\right)-f_{r}\left(t\right)=\frac{1}{2\pi }\left[\frac{d}{dt}\theta \left(t\right)\right]-\frac{1}{2\pi }\left[\frac{d}{dt}\theta \left(t-\tau \right)\right]. \end{array}$$

The resulting beating tone signal is

(5) $$\begin{array}{c} s_{b}\left(t\right)=\frac{\alpha A^{2}}{2}\cos\left[\omega_{0}\tau +2\pi \mu \tau t-\pi \mu \tau^{2}\right], \end{array}$$

the delay for a target located at a distance (range) *R* is $\tau =\frac{2R}{c_{0}}$. The terms consists of the following:

- $\omega_{0}\tau$: The phase shift due to the target distance
- 2πμτ: The beating frequency that can be expressed as
  (6) $$f_{b}=\mu \tau =\frac{2\Delta fR}{T_{M}c}$$
- $\pi \mu \tau^{2}$: The residual video phase, which can be neglected for short distances, if $\pi \mu \tau^{2}\ll \omega_{0}\tau$. For short-range applications of FMCW radars, $\tau \ll \frac{\omega_{0}}{\pi \mu }$, and the residual video phase term can be neglected.

For every FM sweep, the beating tone (5) at the radar output is digitized using an analog-to-digital converter (ADC). Since the beating frequency for a single target is proportional to *R*, for an arbitrary scene containing multiple targets, the mixer output will provide a spectrum of different tones with frequencies proportional to the different target ranges. Therefore, a Fourier transform can be used to isolate and distinguish different targets with a frequency resolution of $\frac{1}{T_{M}}$ and a range resolution of $\Delta R=\frac{c}{2\Delta f}$. This is carried out with a Discrete Fourier Transform (DFT) of the digitized signal as follows:

(7) $$\begin{array}{c} S_{b}\left(k+1\right)=\frac{1}{N_{\mathrm{Smp}}}\sum_{n=0}^{N_{\mathrm{Smp}}-1}s_{b}\left(n+1\right)\cdot \exp\left(-j\frac{2\pi kn}{N_{\mathrm{Smp}}}\right), \end{array}$$

**Figure 1:**
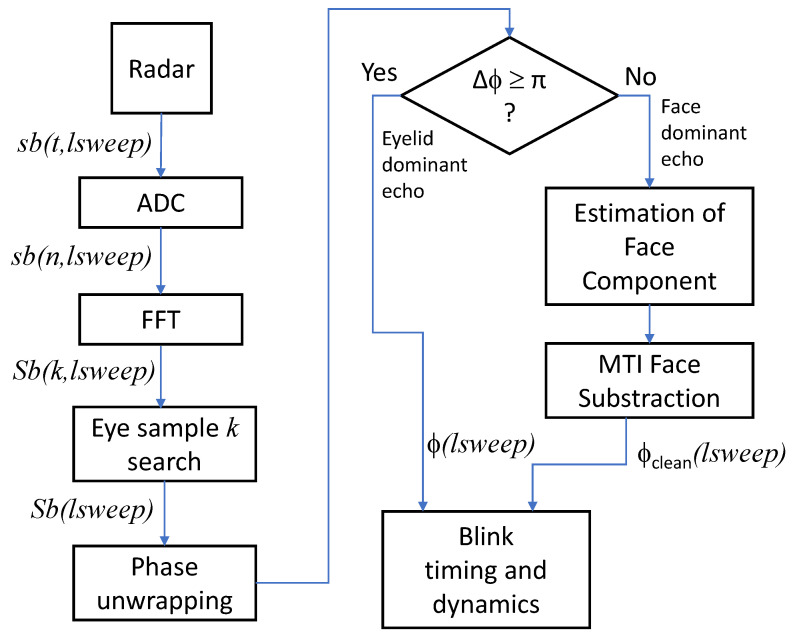
Signal processing algorithm.

To increase the number of samples at the DFT output, the beating tone signal provided by the radar can be extended with zeros before the DFT (zero-padding). The DFT is usually implemented with a Fast Fourier Transform (FFT) algorithm for numerical efficiency reasons [15]. Figure 1 shows a diagram of the signal processing of the applied algorithm, including the phase processing described in Section 5.

The range cell of interest corresponding to the observed body spot is identified and resolved in the frequency domain, and its complex value is extracted and stored for every radar sweep. The micromotion of the body can be ascertained from the changing phase $\phi_{b}\left(k\right)\in \left[0;2\pi \right]$ of the range cell of interest $S_{b}\left(k\right)=abs\left(S_{b}\left(k\right)\right)\cdot \exp\left(j\cdot \phi_{b}\left(k\right)\right)$ along the radar observations. The time between consecutive chirps $T_{\mathrm{obs}}$ is determined by the design of the waveform generator and the bandwidth Δf of the radar signal.

### 2.1. Radar Parameters

**Figure 2:**
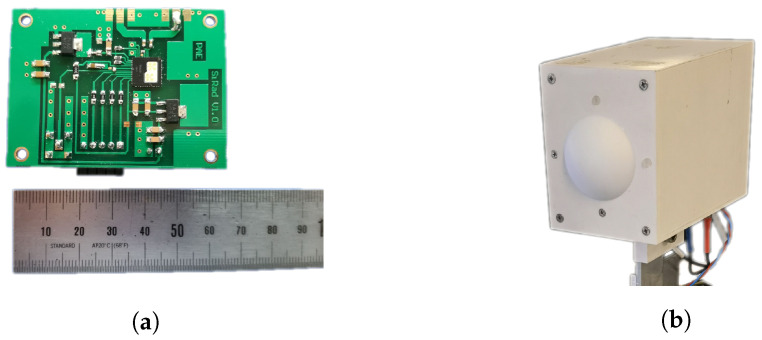
The radar sensor module. The radar front-end is a TRX_120_01 RFE chip mounted onto an FR4 Printed circuit board. (**a**) Circuit board of the radar. (**b**) Prototype of the 120 GHz FMCW radar.

**Figure 3:**
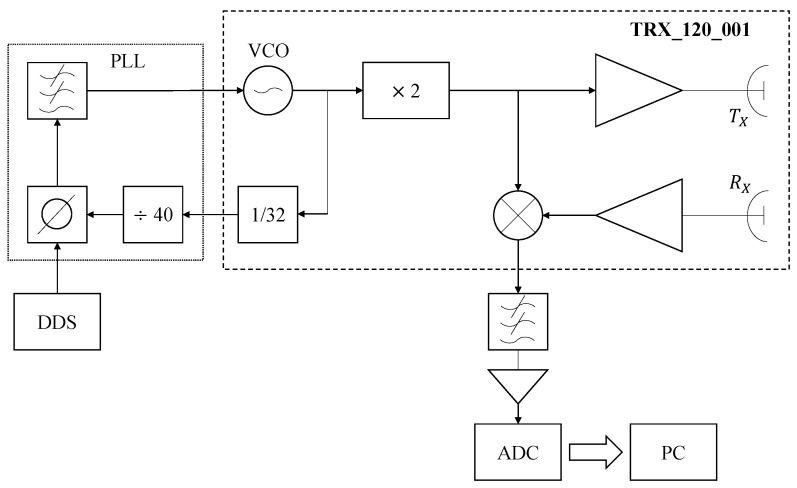
Overview of the radar system. The DDS generates a frequency signal ranging from 47.65 to 48.05 MHz, which is frequency-multiplied through a phase-locked loop (PLL). A voltage-controlled oscillator (VCO) subsequently produces a 61 to 61.5 GHz signal for further frequency multiplication to reach a range of 122 to 123 GHz, which is used as the transmission frequency for observing the target. The echo signal, reflected from the eyelid, is captured by the receiving antenna $R_{X}$, which is then down-converted in a mixer using the transmission signal as a reference. The resulting baseband signal is filtered and amplified, effectively removing high-frequency noise to enhance the signal quality. The received signal is digitized with a 12-bit ADC for further processing and calculation of the eyelid dynamics parameters.

The radar used in this study, shown in Figure 2, was developed in our research laboratory. The system uses a System-on-a-Chip (SoC) 120 GHz radar front-end (TRX_120_001) from [16] feeding a plastic lens antenna. As shown in Figure 3, the instantaneous frequency transmitted by the radar is provided by a Direct Digital Synthesizer (DDS) based on a 1 Gs/s 14-bit DDS chip, programmed as a sawtooth linear FM generator. The local oscillator of the TRX_120_001 is phase-locked to the DDS output, generating a reference submultiple frequency of 1/(64×40) the transmitted frequency in the 120 GHz band. The target range is determined from the frequency peak in the beating tone spectrum. After applying the FFT to the echo signal, and inverting (6), we obtain

(8) $$\begin{array}{c} R=\frac{c}{2}\tau =\frac{T_{M}c}{2\Delta f}f_{b} \end{array}$$

with $f_{b}=\mu \tau$. For a micromotion measurement we evaluate the phase evolution of the range cell of interest containing the target. With $\phi_{b}=-\omega_{0}\tau =-2kR$, we obtain

(9) $$\begin{array}{c} R=\frac{\lambda }{4\pi }\phi_{b}, \end{array}$$

where $\phi_{b}$ is the phase of the beating tone of a backscattering point, and $\lambda =\frac{c}{f_{0}}$ is wavelength corresponding to the transmitted frequency. After detecting the target in a *k* frequency domain sample corresponding to the observed body range *R* resolution cell, the phase and amplitude of this target are evaluated over the entire measurement of *l* chirps (FM sweeps). One single chirp consists of $k=0,\ldots ,N_{\mathrm{Smp}}$ samples. The range determination based on the frequency of the beating tone is not ambiguous if the sampling frequency of the ADC fulfils the Nyquist sampling frequency corresponding to the maximum expected range. The range resolution of a radar operating with a bandwidth Δf is given by

(10) $$\begin{array}{c} \Delta R=\frac{c}{2\Delta f}. \end{array}$$

Since the eyelid motion is small compared to the radar range cell, eyelid monitoring is based on phase rather than range resolution. The cyclic characteristic of the phase introduces a maximum unambiguous phase-based change in range as follows:

(11) $$\begin{array}{c} R_{\mathrm{un}}=\frac{\lambda }{2}. \end{array}$$

For this reason, if the range changes exceed $R_{\mathrm{un}}$, the phase must be unwrapped to obtain the correct displacement.

Table 1 shows the radar parameters used for this study.

### 2.2. Assessment of the Radar’s Safety

The IEEE, in [17], defines exposure limits for electric, magnetic, and electromagnetic fields within 0 Hz to 300 GHz. It is important to respect these safety limits to minimize health effects and risks during measurements and interference with other radio frequency-based equipment. In this study, the radar specifications and lens characteristics allow us to estimate a radiated power of −4.6 dBm (0.35 mW) and an illuminated spot diameter of 3.5 cm with a −3 dB contour at a typical operation range of 1 m. The resulting illumination power density is 36 μW/cm^2^, well below the 2.3 mW/cm^2^ ERL of local exposure limits at 120 GHz recommended in [17] for people in unrestricted environments, which is the most stringent limit. For this reason, the proposed technique can be considered to be of minimal health risk, with exposure limits well below the exposure limit recommendations.

## 3. Simulations

**Figure 4:**
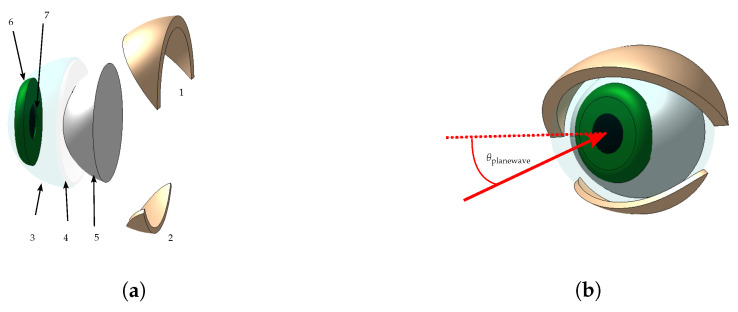
Approximated model of the anterior part of the eye. (**a**) Components of the eye model: (1) Upper eyelid, which will be closed in the simulation; (2) lower eyelid; (3) cornea; (4) anterior chamber; (5) sclera; (6) iris; and (7) pupils. (**b**) Front view of the eye model.

Electromagnetic simulations on a simplified dielectric model of a human eye were carried out to predict and interpret the radar backscattering experimental results. Figure 4 shows the model used in the evaluation of the radar backscattering. The eyeball was modeled as a dielectric sphere with a radius of 13 mm, a relative permittivity $\varepsilon_{r}=10$, and a loss tangent tanδ=0.8. A homogeneous permittivity sphere was assumed since the cornea, sclera, and iris have similar values of permittivity. The model values were obtained from the Foundation for Research on Information Technologies in Society (IT’IS), which provided the tissue properties of the eye components [18]. The lower and upper eyelids covering the eyeball were modeled with dielectric layers of a 2 and 3 mm thickness, respectively [19], with a relative permittivity of $\varepsilon_{r}=8$ and tanδ=0.647. The simulations were carried out using CST Microwave Studio.

**Figure 5:**
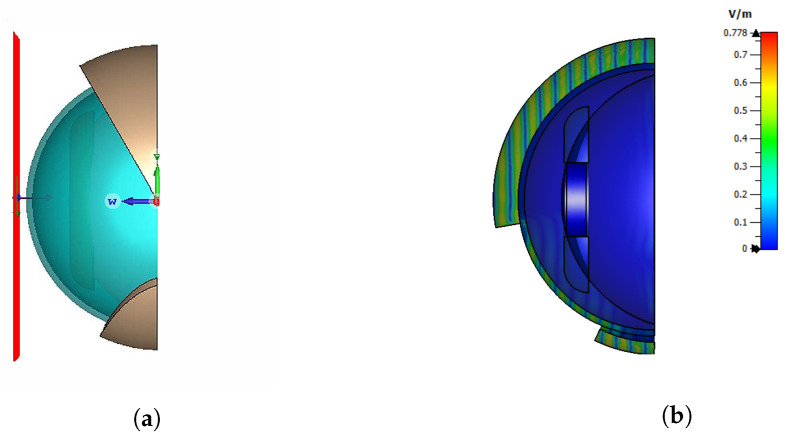
Eye with lid model in Microwave Studio. (**a**) A horizontal plane wave is directed towards the eye. (**b**) Electric field amplitude in the eye model.

**Figure 6:**
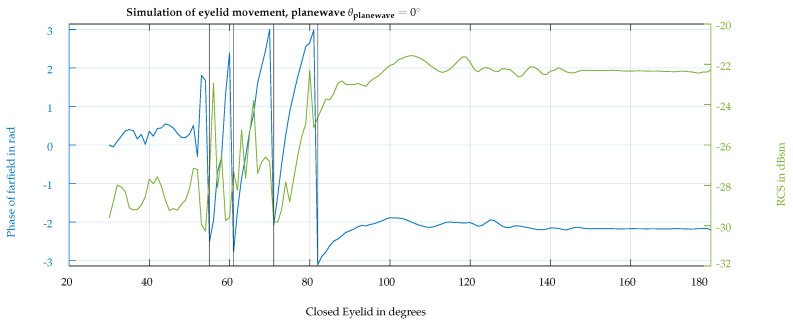
Phase (blue line) and RCS (green line) of the backscattering simulation results with respect to the eyelid closure angle from open ($20^{{}^{\circ}}$) to complete closure ($180^{{}^{\circ}}$).

To capture the eyelid edge from different viewing angles, the upper eyelid used in the simulations has a thickness of 3 mm. In Figure 5a, a plane wave is illuminating the eye surface with the propagation vector directed horizontally towards it. The incidence angle $\theta_{\mathrm{planewave}}$ is also visualized in Figure 4b. The upper eyelid has a $30^{{}^{\circ}}$ angle with respect to the vertical axis in this figure. In subsequent simulations, the eyelid closes until it reaches $180^{{}^{\circ}}$. The strong attenuation caused by the eye with a closed eyelid on the incoming field is shown in Figure 5b. Consequently, the penetration depth of the eye tissues is very small, and the eye components behind the cornea have a negligible influence on the radar measurement. Figure 6 displays the simulated phase and radar cross-section (RCS) during the eyelid closure progression, expressed as the eyelid angle. A noticeable oscillation in the RCS is attributed to the superposition of changing phase reflections from the eye and eyelid, dependent on the angle of the eyelid’s closure. The phase rotates more than $360^{{}^{\circ}}$ with an eye closure angle of $55^{{}^{\circ}}$ to $85^{{}^{\circ}}$, with the transition of rotation from +π to −π shown by the vertical black lines. According to (9), a phase difference $\Delta \phi_{b}=4\times 2\pi -2.2$ corresponds to a relative change in the distance of the object from the radar of ΔR=4.5 mm. When the closure angle is higher than $90^{{}^{\circ}}$, no significant backscattering changes are observed since the frontal area of the eye contributing to backscattering is stable.

## 4. Experimental Validation

The lens in front of the radar TRX_120_01 SoC produces a narrow spot about 3.5 cm in diameter at a radar distance of 1 m. The ranging radar’s performance was validated with a small 2 cm side corner reflector controlled with a micrometric positioner. The theoretical parameters of the range resolution and phase/range sensitivity were confirmed experimentally. With a displacement in phase sensitivity of 3.4 μm per degree, the capability to determine the range with submillimetric precision has been validated.

**Figure 7:**
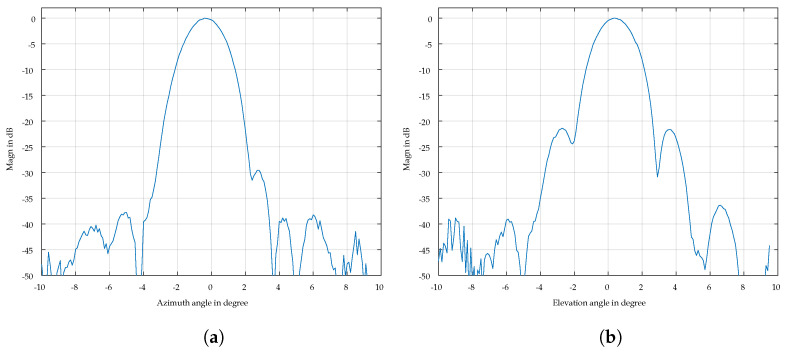
Azimuth and elevation cuts of the antenna radiation pattern. (**a**) Azimuth cut. (**b**) Elevation cut.

The pattern of the lens antenna was measured using a static small corner surrounded by absorbing material as the reference target and scanning the radar antenna both in azimuth and elevation with a dual-axis angular positioner. Figure 7 shows the antenna pattern in Cartesian coordinates. The 3 dB angle is approximately $\theta_{\mathrm{beam},3\mathrm{dB}}=2^{{}^{\circ}}$ in the horizontal and vertical direction. The side lobes are suppressed to −33 dB.

## 5. Eyelid Results

### 5.1. Radar Pointing Horizontally

Taking into account the ADC board’s memory limitations, the radar was configured to acquire echo beating tone signals along 8000 consecutive chirps. With a chirp repetition period of 3 ms, the total acquisition time per observation was 24 s, which is enough to evaluate the performance of the proposed eye blink detection and eyelid dynamics characterization.

**Figure 8:**
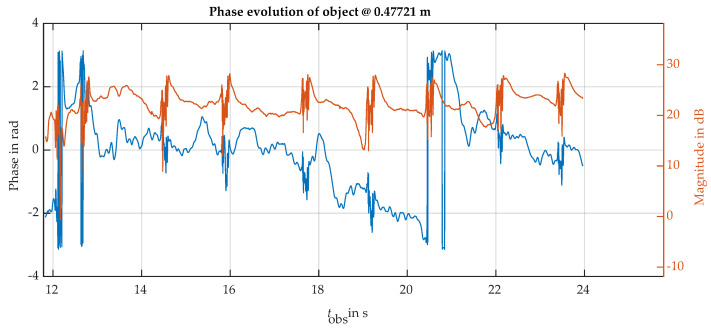
Phase (blue line) and magnitude (red line) of the eye spot backscattering. The radar is located at a 1 m distance in front of the subject’s face, observing the eye area with a horizontal beam. Several blinks can be observed, producing fast magnitude and phase oscillatory transients.

The initial eyelid dynamics measurements were conducted with the radar beam aligned horizontally with the eye axis. Precise alignment of the radar was necessary due to the narrow beam of the antenna. A small corner reflector was used to confirm the correct pointing of the radar. Figure 8 shows the magnitude and phase of the resolution cell backscattering evolution corresponding to the observed eye. Several blink events can be identified as short and fast transient oscillations, which are present in both the magnitudes and the phase of the observed backscattering. The phase’s slow negative slope trend reflects a small movement made by the subject head changing the target-radar range along the measurement window of 24 s. Phase unwrapping is needed to correctly recover the target motion to avoid the phase jumps observed close to the ±π phase limits. After phase unwrapping, the value of the phase change peaks can be used to detect the blink events and determine the average blink period and the period dispersion or irregularity.

**Figure 9:**
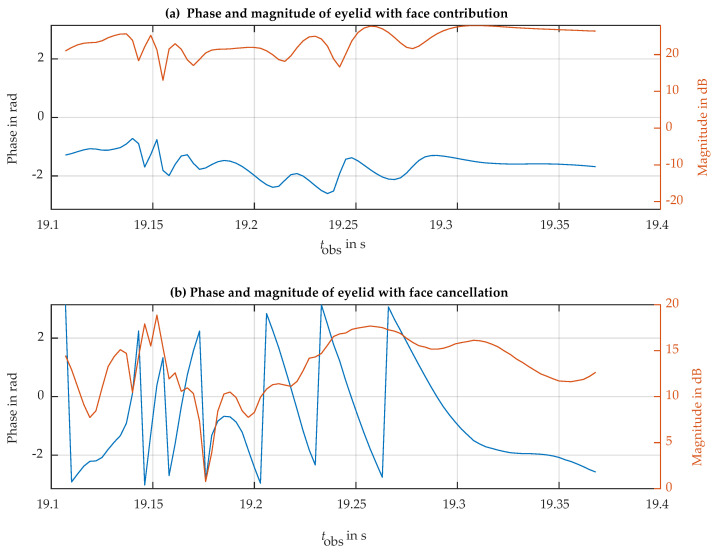
Detail of the phase (blue line) and magnitude (red line) of the eye spot backscattering shown in Figure 8 at the time of one of the blinks. Before (**a**) and after (**b**) face background subtraction.

Figure 9a shows the magnitude and phase of one of the blinks. The ripple in magnitude indicates an interference pattern between two or more scattering centers in the same resolution cell with different phase rotation paces. To reduce the impact of the face interference on the eyelid radar observations, we have used a two-scattering-center model. One of the scatterers is the fast-moving eyelid, with a fast phase rotation linked to blink closure and aperture. The second scatterer models the rest of the observed face, with slower magnitude and phase dynamics. Under this model, every transition between the local maxima and minima corresponds to a half-cycle relative phase turn (180 degrees) in the eyelid phase with respect to the unwanted background of the face. The fact that the phase in the upper part in Figure 9a rotates only with fractions of a cycle indicates that the scattering of the face is dominant and the scattering of the eyelid has a smaller magnitude. Exploiting this model, the face contribution to the radar echo signal can be obtained from the evolution of maximum and minimum magnitude. For example, in both the maxima and minima samples, the phase of the observation corresponds to the phase of the dominant face background. The magnitude of the unwanted background can be obtained from the average value of consecutive maxima and minima magnitudes. This information was used to estimate the background face phasor and its evolution along each blink event. The lower part in Figure 9b shows the same blink event after subtracting the estimated contribution of the face background. This technique can be considered an adaptive extension of the classical Moving Target Indicator technique common in pulsed radars for rejecting unwanted static clutter by cancellation [20]. After background cancellation, the phase shows the expected multi-turn evolution, reproducing the eyelid motion in detail, in a similar way that was predicted by the numerical simulation. Note that the eye closes faster than it opens; in the closure step, the eyelid covers the cornea, implying a shorter radar range, which translates into a positive phase slope, and the opposite occurs for the aperture motion of a blink.

### 5.2. Slanted Observations

**Figure 10:**
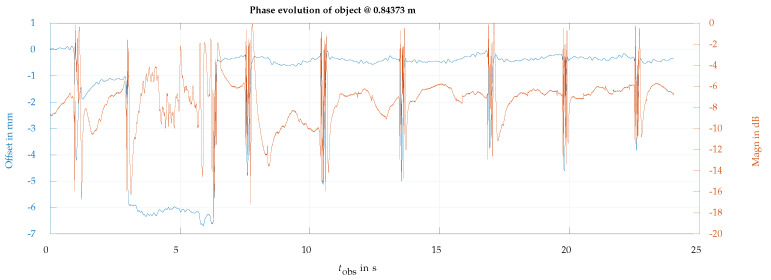
Phase (blue line) and magnitude (red line) of the eye backscattering using an ascending angle of observation of $55^{{}^{\circ}}$ with respect to the horizontal direction. Blinking movements are detected at approximately 1 s, 7.6 s, 10.5 s, 13.5 s, 16.9 s, 19.7 s, and 22.5 s. The phase evolution clearly reflects the interval of the eyelid voluntary motion, closing and opening at 2.9 s and 6.4 s, respectively.

**Figure 11:**
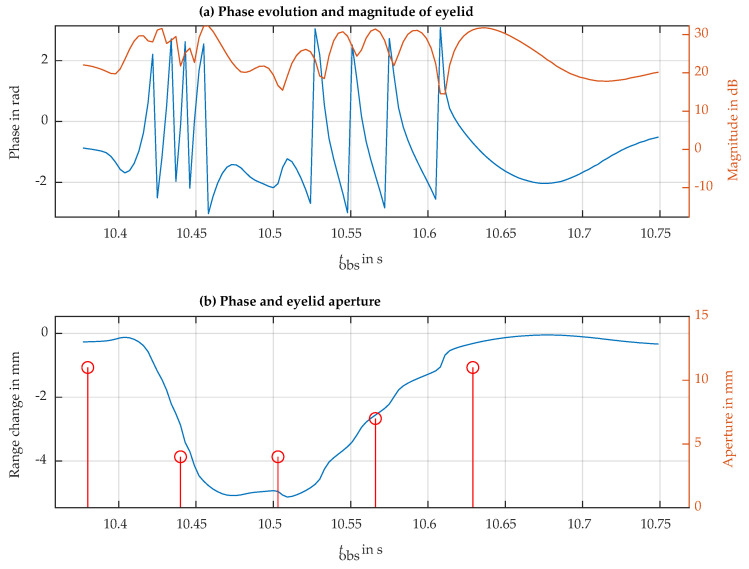
The blink signal at approximately 10.5 s in Figure 10. (**a**) Phase evolution (blue line) and magnitude (red line). (**b**) Phase of the blink process (blue line) with the sampling data of the eye aperture (red line) during this process.

Another way to reduce the background contribution of the face is to observe the eye at a slanted angle. In our case, the radar was relocated below the face level, observing the eye with a slanted antenna beam pointing upwards with an angle of $55^{{}^{\circ}}$ with respect to the horizontal direction. In this slanted direction of observation, the parts of the face orthogonal to the radar observation are reduced up to the point that the face’s contribution to the measured echo is no longer dominant. Under this condition, the phase rotation of the eyelid can be observed without requiring background cancellation. Figure 10 shows a complete recording of 24 s, where a blink can be observed at t=1 s, the eye is voluntarily closed in the interval of 3 s to 6 s, and this is followed by six normal blinks until the recording ends, for t=24 s. The phase term was converted into a relative range offset in mm. The measurements indicate a typical closure range offset of nearly 5 mm, which is likely influenced by the movement of the skin around the eye. In Figure 11, it is possible to observe one blink in more detail. Based on the details observed in this measurement, we can determine the average blinking period using Figure 10 and examine the dynamics of a single blink using Figure 11. For instance, the average blinking period and standard deviation were calculated using the last six blinks measured in the subject, and additional dynamics parameters are presented in Table 2. Furthermore, a fast-speed video was recorded in synchrony with the radar acquisition. Slow-motion video reproduction allowed us to relate the video-recorded eyelid closure/aperture timing with the timings of the dynamics parameters obtained from the radar observations in order to validate the proposed technique.

### 5.3. Errors in the Instrument and Methodology

Ranging errors in the radar instrument are caused by the phase noise present in the echo signal of the eyelid. In the typical eyelid observation conditions of range and Signal-to-Noise, the standard deviation of the phase noise is $\sigma_{0}=0.0024$ rad, which is equivalent to a range estimation error of $\sigma_{r}=\frac{\sigma_{r}\lambda_{0}}{4\pi }=0.462\;\mu m$. This is the intrinsic radar instrument error.

However, the eyelid is not measured in isolation. The interference created by the subject’s face introduces additional range deviations well above the instrumental error. These deviations can be observed as a phase ripple in Figure 11, which introduces an estimated phase-typical deviation of $\sigma_{\mathrm{measurement}}=0.1481$ rad, corresponding to a range-typical deviation of $\sigma_{u}=28.486\;\mu m$.

## 6. Discussion

It is important to explore the impact of various circumstances on signal processing and examine the feasibility of extracting data for the successful detection of eyelid blinks in different environments. In the previous measurements, the sweep bandwidth was set to 3 GHz and was later reduced to 1 GHz by applying a tapered time domain window to the beating tone of the radar. As a result, the resolution cell in the direction of propagation, defined by the bandwidth Δf, increased from 5 cm to 15 cm. With accurate alignment and cancellation of the face background, no substantial loss of information occurred due to a reduction in bandwidth.

In recent years, the majority of the research on eyelid detection has relied on optical and electromagnetic methods. Camera-based algorithms, however, often face challenges, including factors such as facial features, posture, lighting conditions, the angle of view of the lens, and distance from the camera. For instance, the advanced algorithm proposed in [21] is highly dependent on a face detection system in capturing the subject’s face. Additionally, the algorithm exhibits sensitivity to the distance of the subject, and its performance is significantly hindered when the subject’s eyes’ natural colors and contours are altered with makeup. The method proposed in [22] for monitoring eye movements using electromagnetic sensing technology requires the inclusion of magnetic materials into the eyelashes, which may pose potential risks and inconvenience. In contrast, the above issues can be effectively mitigated using the radar technique proposed, which offer robustness to illumination conditions without requiring any kind of contact or eye manipulation. Meanwhile, it was tested whether wearing glasses would affect the measurement of eyelid movement. This is an important consideration if a patient that uses glasses needs to be monitored. Wearing glasses did not have a significant impact on the results.

It is worth noting that certain limitations may affect the use of radar technology to measure eyelid dynamics signals. In our experiments, the measurements were conducted in a controlled indoor environment that was clean, static, and free from complex interference. Consequently, external noise and environmental factors did not significantly impact the measurement accuracy or stability. However, in more complex scenarios, such as monitoring drivers, the robustness of the algorithm may be compromised. The small size of the eyelid as a target, combined with the potential movements of the subject, introduces challenges in terms of the radar’s precision in target alignment. This difficulty in maintaining accurate radar pointing could reduce the reliability in complex cases.

To address these limitations, it is possible to explore the integration of multi-input, multi-output radar technology in future work, which has the potential to enhance the spatial resolution and target tracking, thereby improving robustness in challenging environments and reducing the impact of movement of the subject on the accuracy of measuring the eyelid dynamics.

## 7. Conclusions

In summary, this study confirms that using radar to detect and characterize eye blink dynamics is an alternative to using high-speed cameras or magnetic coils, avoiding the need for good illumination requirements or contact with the patient. The fine beam width and bandwidth of the FMCW radar can isolate the observation area from other parts of the body and surrounding objects. At 120 GHz, the depth of penetration into biological tissues is negligible. For this reason, the measured backscattering changes reflect the alterations in the shape/geometry of the observed body surface within the antenna beam spot. Depending on the direction of observation, the eyelid return may also be affected by echoes from the surrounding face areas, reducing the phase sensitivity of the echo with respect to the eyelid dynamics. A background cancellation technique has been proposed, showing the capability to recover the eyelid echo phase in these situations. Alternatively, observing the eye at a slanted angle with respect to the frontal face direction can also minimize the unwanted face interference. This study also tested the impact of reducing the radar bandwidth to 1 GHz and glasses being worn during measurement, without relevant impacts on the results obtained. Upgrading the radar with a beam-steering capability, for example, using a multiple-input, multiple-output configuration, would allow us to track the eye area automatically, allowing for more robust operation in front-of-body motion resulting from activities such as driving a vehicle.

## Figures and Tables

**Table 1:** System parameter for simulations and measurements.

Parameter	Calculation	Value
Center frequency	$f_{0}$	122.5 GHz
Bandwidth	Δf	1–3 GHz
Sweep time	$T_{M}$	1.5 ms
Slope	$\mu =\frac{\Delta f}{T_{M}}$	(1–3) GHz/1.5 ms
Sweep repetition period	SRP	3 ms

**Table 2:** Extracted time characteristics of the eye blink at $t_{\mathrm{obs}}=10.5$ s in Figure 10 and Figure 11. Dynamics of different blinks, like closing speed, for example, can be generated from such data. The average blink period and the standard deviation in the blink period for the whole acquisition are also shown.

Parameter	Start	End	Duration
Duration	$t_{\mathrm{obs}}=10.40$	$t_{\mathrm{obs}}=10.70$	300 ms
Closing	$t_{\mathrm{obs}}=10.40$	$t_{\mathrm{obs}}=10.47$	70 ms
Closed eyes	$t_{\mathrm{obs}}=10.47$	$t_{\mathrm{obs}}=10.51$	40 ms
Opening	$t_{\mathrm{obs}}=10.51$	$t_{\mathrm{obs}}=10.70$	190 ms
$\bar{T}=2.99$ s	$\sigma_{T}=238$ ms		

## Data Availability

The experimental data obtained in this work is available on request from corresponding author due to lack of suitable data repository at this time.
